# The presence of high mobility group box-1 and soluble receptor for advanced glycation end-products in juvenile idiopathic arthritis and juvenile systemic lupus erythematosus

**DOI:** 10.1186/1546-0096-12-50

**Published:** 2014-12-03

**Authors:** Dubravka Bobek, Danka Grčević, Nataša Kovačić, Ivan Krešimir Lukić, Marija Jelušić

**Affiliations:** Department of Pediatrics, Division of Pediatric Rheumatology and Immunology, University Hospital Centre Zagreb, University of Zagreb School of Medicine, Kišpatićeva 12, 10000 Zagreb, Croatia; Department of Physiology and Immunology, University of Zagreb School of Medicine, Zagreb, Croatia; Department of Anatomy, University of Zagreb School of Medicine, Zagreb, Croatia; Department of Research in Biomedicine, and Health, University of Split School of Medicine, Split, Croatia

**Keywords:** Juvenile idiopathic arthritis, Juvenile systemic lupus erythematosus, High mobility group box-1, Solubile receptor for advanced glycation end-products

## Abstract

**Background:**

The involvement of high mobility group box-1 (HMGB1) in various inflammatory and autoimmune diseases has been documented but clinical trials on the contribution of this pro-inflammatory alarmin in children with juvenile idiopathic arthritis (JIA) and systemic lupus erythematosus (SLE) are basically absent. To address the presence of HMGB1 and a soluble receptor for advanced glycation end products (sRAGE) in different subtypes of JIA and additionally in children with SLE, we enrolled a consecutive sample of children harvested peripheral blood as well as synovial fluids (SF) at diagnosis and correlated it with ordinary acute-phase reactants and clinical markers.

**Methods:**

Serum and synovial fluids levels of HMGB1 and sRAGE in total of 144 children (97 with JIA, 19 with SLE and 27 healthy controls) were determined by ELISA.

**Results:**

The children with JIA and those with SLE were characterised by significantly higher serum levels of HMGB1 and significantly lower sRAGE levels compared to the healthy controls. A positive correlation between serum HMGB1 and ESR, CRP, α2 globulin was found while serum sRAGE levels were inversely correlated with the same inflammatory markers in children with JIA. Additionally, high level of serum HMGB1 was related to hepatosplenomegaly or serositis in systemic onset JIA.

**Conclusion:**

The inverse relationship of the HMGB1 and its soluble receptor RAGE in the blood and SF indicates that inflammation triggered by alarmins may play a role in pathogenesis of JIA as well as SLE. HMGB1 may serve as an inflammatory marker and a potential target of biological therapy in these patients. Further studies need to show whether the determination of HMGB1 levels in patients with JIA can be a useful guideline for detecting disease activity.

## Background

Juvenile idiopathic arthritis (JIA) is a heterogeneous group of arthritis diseases with the onset before 16 years of age and persistent for more than 6 weeks. Systemic JIA (sJIA) is quite distinct from oligoarticular or polyarticular subtypes with the prominence of systemic and inflammatory features [1]. At the initial presentation of sJIA, arthritis may not be present. Hence, in children with sJIA, the illness cannot be discriminated from systemic infections by clinical or laboratory parameters. As a result, early initiation of suitable antiinflammatory therapy is difficult [2]. In the field of pediatrics, in addition to sJIA, there is childhood SLE (cSLE) – is another acute inflammatory disorder frequently presenting a diagnostic and therapeutic challenge. cSLE is a complex autoimmune inflammatory disease characterized by multiorgan involvement. Approximately 20% of SLE cases begin before age 19 and often have a poor prognosis [3].

Although the pathogenesis of sJIA and cSLE remains unclear, a novel studies seem to point in the direction of innate, rather than adaptive immune response [4, 5]. As shown during the past couple of decades, activation of innate immune system seems to rely on a special class of molecules, namely damage-associated molecular patterns (DAMP) or alarmins [6–10]. A prototype of alarmins, high-mobility group box-1 (HMGB1), released to the extracellular space from activated or necrotic cells, acts as a key molecule of innate immunity [11, 12]. Recently, Garcia-Romo and collegues have demonstrated that in cSLE, HMGB1 is released during NETosis, a form of regulated cell death that occurs with neutrophils (neutrophil extracellular traps, NET). As it has been shown in this study, HMGB1 itself can also induce NETosis and play an important role as a proinflammatory mediator in cSLE [13].

According to previous studies, extracellular HMGB1 can amplify inflammation and enhance immune responses by interacting with the receptor for advanced glycation end-products (RAGE) [14–20]. Although being the first receptor demonstrated to bind HMGB1, some members of the Toll-like receptor (TLR) family, such as TLR2, TLR4, TLR9 and chemokine receptor C-X-C motif receptor 4 (CXCR4) can bind HMGB1, too [21, 22]. Transmembrane RAGE on monocytes, macrophages and other cells is overexpressed in response to increasing concentration of its ligand HMGB1 and triggers inflammatory immune response. Moreover, RAGE also exists in a soluble form (sRAGE), which mitigates proinflammatory effects of HMGB1, as shown by several studies [23–25]. It is therefore hypothesized that sRAGE binds RAGE-ligands, thus acting as a “decoy” receptor [10].

Although data coming from clinical studies on children are still very few in number, the presence of antibodies to HMGB1 in JIA patients is documented [26, 27]. Furthermore, recent research has showed high levels of HMGB1 in synovial fluid in children with JIA correlating with early onset of disease [28]. In addition, there is one in vivo study in juvenile SLE patients that has shown a correlation of HMGB1 with the disease activity regardless of nephritis presence [29]. Nevertheless, the role of HMGB1 in JIA and cSLE has not been elucidated yet.

The aim of the present study was to determine the presence of HMGB1 and sRAGE in sera and synovial fluids in children with the different subtypes of JIA and juvenile SLE, in order to assess their possible association to clinical and serum markers for inflammation.

## Methods

The study was designed as a prospective trial in which a total of 144 children were enrolled. Among 97 patients with newly diagnosed JIA, 35 children had polyarticular type, 34 oligoarticular, and 27 systemic type of the disease. All JIA patients fulfilled the ILAR diagnostic criteria [30]. Furthermore, 19 children with newly diagnosed SLE were included in this study, each fulfilling the American College of Rheumatology diagnostic criteria for SLE [31], were included in this study. The control groups included 28 children admitted to University Hospital Centre Zagreb either for reasons not related to autoimmunity or infectious diseases or for surgery not in connection with any inflammatory diseases.

All the children were patients of Division of Pediatric Rheumatology and Immunology, University Hospital Centre Zagreb between 2007 and 2012. The study was approved by the Ethics Committee of the Zagreb University School of Medicine and parental informed consent was obtained for each child. For all patients, the following routine laboratory tests were performed at the Department of Clinical Laboratory Diagnostics, University of Zagreb School of Medicine: erythrocyte sedimentation rate (ESR; mm/hour), C-reactive protein levels (CRP; mg/l), α2-globulins (%), γ-globulins (%), white blood cells count (WBC; ×10^^^9/l), red blood cells (RBC; ×10^^^12/l), platelets (PLT; ×10^^^9/l), hemoglobin (g/l), haematocrit (%). For patients with SLE autoantibodies, rheumatoid factor (RF), and C3 and C4 complement components levels were also analysed.

The blood samples were collected at the time of diagnosis, prior to the initiation of immunosuppressive treatment. Seven patients did not receive any drug, while the rest of the patients received nonsteroidal anti-inflammatory drugs. The clinical and demographic data were recorded at the time of the blood collection.

Venous blood was extracted in test tubes, which contained an anticoagulant (EDTA), always between 9 and 10 a.m. Aliquots of the peripheral blood were centrifuged at 3500 rpm for 5 minutes at room temperature, after which the supernatant is aspirated and stored in kriotube (Nunc, Denmark) at -70°C until used. The serum levels of alarmins were determined with commercially available enzyme-linked immunosorbent assay (ELISA) kits: RAGE (R&D Systems, Minneapolis (MN), USA) and HMGB1 (Shino-Test Corporation, Tokyo, Japan) according to the manufacturer’s protocol. In addition, levels of HMGB1 and sRAGE were determined by the same kits in synovial fluid of 21 patients with oligoarticular and 7 patients with polyarticular when there was an indication for evacuation of the synovial fluid.

All statistical analyses were performed with R statistical programming language [32]. Differences between groups of patients and the control group were analysed by Kruskal-Wallis test, followed by a post-hoc test of patient groups against the control group [33]. Correlations between variables were analysed with the Spearman’s correlation test. P-values were adjusted with Bonferroni method for multiple correlations between HMBG1 and sRAGE and clinical variables. A two-tailed *p*-value <0.05 was considered as statistically significant. ROC curves were constructed with the help of pROC package for R statistical programming language [34]. On scatterplots, a smoothing line of locally weighted polynomial regression (*loess*) was added [35]. Levels of HMGB1 serum in patients with hepatosplenomegaly and serositis (within the systemic JIA group) and with lupus nephritis (within the SLE group) were compared with patients without corresponding features within groups by the Mann–Whitney U test.

## Results

### Characteristics of patients with JIA, SLE and controls

Basic laboratory findings of the children with JIA, SLE and healthy controls recruited in the study are shown in Table 1.

**Table 1:** **Laboratory characteristics of children with juvenile idiopathic arthritis (JIA), systemic lupus erythematosus (SLE) and healthy controls**

Characteristic	***p***-value	Juvenile idiopathic arthritis			SLE (n = 19)	Healthy controls (n = 28)
		Systemic (n = 27)	Oligoarticular (n = 34)	Polyarticular (n = 35)		
No. girls/boys	-	7/20	27/7	27/8	14/5	20/8
Age, years	-	9.9* (5.3-12.2)	2.9* (0.4-8.2)	6.4 (7.2-12.3)	14.4* (10.6-15.9)	6.2 (0.9-11.9)
HMGB1 (pg/ml)	< 0.001	17402.50* (6400.57-80844.98)	3551.97* (108.89-10713.20)	4374.04* (273.50-93493.88)	8523.92* (3639.35-98296.88)	1149.70 (10.89-1976.47)
sRAGE (pg/ml)	0.006	1237.53* (46.42-6634.4)	2269.84 (225.60-5137.92)	1757.76 (230.40-4730.88)	924.45* (118.08-10569.20)	2275.80 (162.37-9206.38)
ESR (mm/h)	< 0.001	86* (34–139)	17* (5–41)	40* (5–140)	57* (10–113)	7 (2–36)
CRP (mg/l)	< 0.001	145.0* (6.9-264.0)	2.6 (0.2-25.7)	23.5* (0.1-156.0)	3.0 (0.6-25.0)	2.0 (0.1-6.8)
Hb (g/l)	< 0.001	111* (80–128)	123 (99–144)	115* (75–140)	119 (78–140)	124 (110–145)
Hct (%)	< 0.001	0.34* (0.23-0.56)	0.37 (0.29-0.43)	0.35 (0.24-0.43)	0.36 (0.22-0.42)	0.37 (0.32-0.52)
PLT, ×10^^^9/l	< 0.001	397* (125–563)	391 (180–651)	363 (131–1038)	222 (122–568)	322 (176–414)
RBC, ×10^^^12/l	< 0.001	4.17* (3.30-4.96)	4.51 (3.54-5.31)	4.26* (3.60-5.88)	4.13* (2.88-5.09)	4.63 (4.10-5.17)
WBC, ×10^^^9/l	< 0.001	9.0* (6.4-29.9)	8.7* (4.0-17.1)	8.5* (3.5-27.0)	4.3 (2.0-19.4)	7.4 (2.5-9.7)
γ-globulin (%)	< 0.001	17.0* (7.8-39.0)	14.2 (7.9-21.5)	21.8* (10.0-32.1)	22.2* (12.0-38.2)	13.9 (6.8-19.2)
α2-globulin (%)	< 0.001	15.3* (11.2-26.3)	9.9* (5.2-16.7)	13.5* (12.9-19.2)	10.3* (8.0-17.7)	7.2 (4.9-14.3)
ANA (RU/ml)	-				640 (100–10242)	
anti-dsDNA (RU/ml)	-				285 (68–2480)	
C3 (mg/l)	-				0.87 (0.32-1.74)	
C4 (mg/l)	-				0.13 (0.03-0.32)	

*Significantly different from the healthy controls (Kruskal-Wallis test followed by a post-hoc test). ESR = erythrocyte sedimentation rate; CRP = C-reactive protein; Hb = hemoglobin; Hct = haematocrit; PLT = platelets; RBC = red blood cells; WBC = white blood cells; ANA = antinuclear antibody; anti-dsDNA = double stranded DNA antibodies; C3,C4 = complement components.

The data are summarized as median and range.

During the initial examination, all the patients with systemic-onset JIA presented with spiking fevers and skin rash as well as high ESR, CRP and α2-globulins. Twelve JIA patients presented with arthritis, at disease presentation. The rest of the patients with sJIA developed arthritis later on while being hospitalised. Ten patients presented with lymphadenopathy, seven with hepatosplenomegaly and three with serositis. All the patients with SLE were antinuclear antibody (ANA) positive, 87% had double stranded DNA antibodies (anti-dsDNA), 63% anti-Smith (Sm), 25% anti-SSA, 19% anti-SSB, 26% anti-ribonucleoprotein (RNP) antibodies, 69% anti-histones, but none of the patients were positive for rheumatoid factor (RF). Six SLE patients were presented with constitutional symptoms (fever, fatigue and weight loss), five with malar rash, four with renal involvement, and two with polyarthritis (data not show).

### The inverse relationship of the HMGB1 to sRAGE in blood samples from children with JIA and SLE taken at diagnosis

Serum levels of HMGB1 were significantly higher (P < 0.001) in children with three major types of JIA and in children with SLE, compared to healthy controls (Table 1).

**Figure 1:**
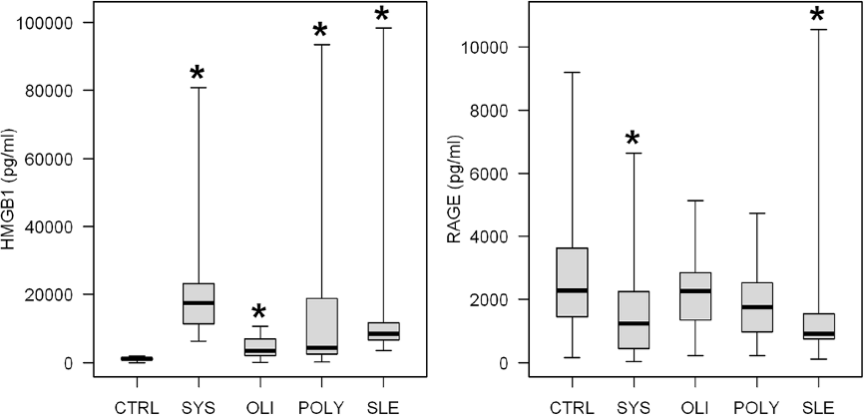
**Serum HMGB1 and sRAGE levels in patients and healthy controls.** Serum levels of high mobility group box-1 (HMGB1) and soluble receptor for advanced glycation endproducts (sRAGE) between children with three major subgroups of juvenile idiopathic arthritis (JIA), systemic lupus erythematosus (SLE) and healthy controls. Boxes represent the interquartile range, the thick horizontal line within the boxes represents the median, and the whiskers extend to the most extreme data points (range).

As shown in Figure 1 the median serum HMGB1 levels in children with systemic-onset JIA (17402 pg/ml) were 15 fold higher than those in control group (1149 pg/ml) and were also significantly higher than those in children with oligoarticular (3552 pg/ml), polyarticular (4374 pg/ml) or SLE children (P < 0.05). There was no statistical difference of the median serum HMGB1 levels between children with oligoarticular and those with polyarticular onset JIA.

When the association between HMGB1 serum levels and clinical characteristics of the disease was tested, we found that systemic JIA children with hepatosplenomegaly and serositis (27695,03 pg/ml) had significantly higher HMGB1 serum levels compared with patients without (12678,06 pg/ml) corresponding features (P < 0.001). Next, in a group with SLE children, we had four patients presenting with lupus nephritis who also had higher levels of HMGB1 (the median concentrations were 36372,97 and 7013,95 pg/ml). The difference was significant (P < 0.003).

At the same time, compared to the healthy children, the children with all subgroups of JIA as well as children with SLE were characterized by lower serum levels of sRAGE, but the difference was significant only in patients with systemic-onset of JIA and in children with SLE (Table 1).

**Figure 2:**
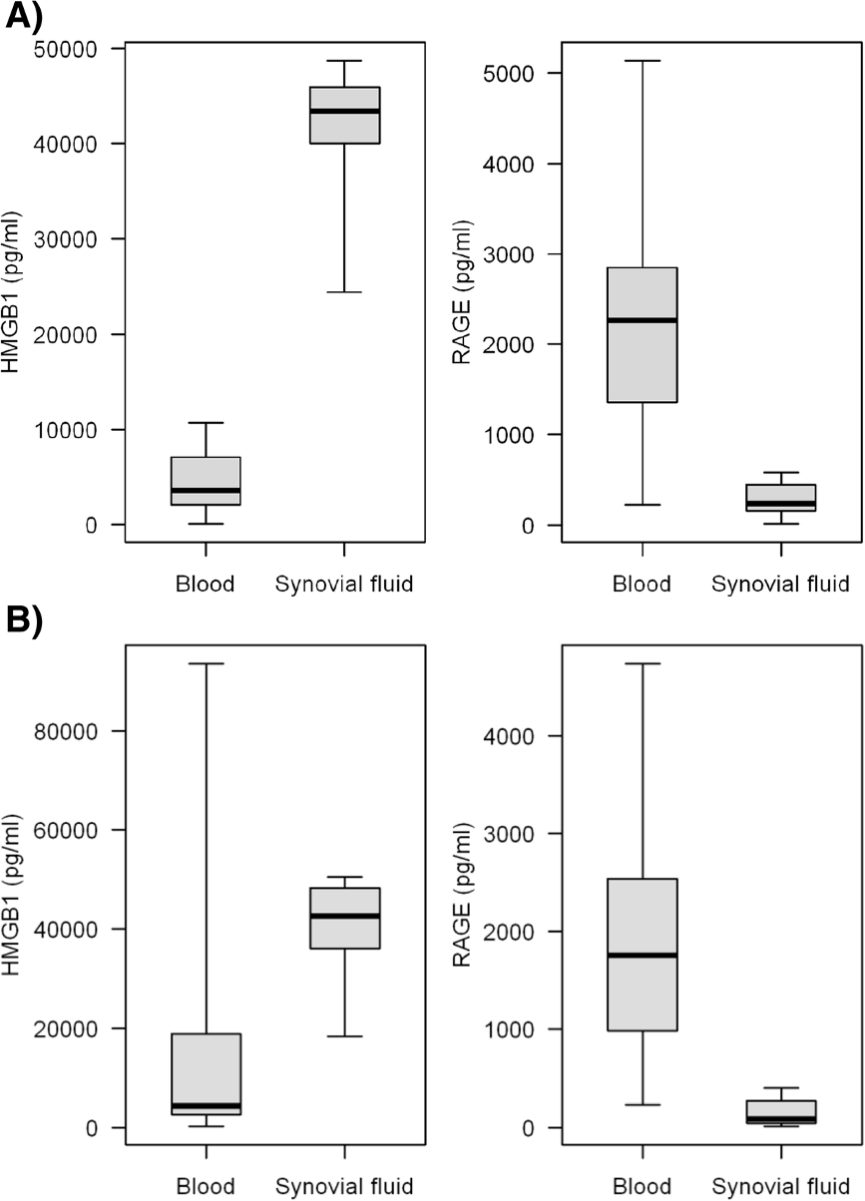
**The median concentrations of synovial fluid HMGB1 in patients with oligoarticular and polyarticular JIA.** Significantly increased high mobility group box-1 (HMGB1) and significantly reduced soluble receptor for advanced glycation endproducts (sRAGE) levels in synovial fluid to blood samples from patients with oligoarticular **(A)** and polyarticular **(B)** juvenile idiopathic arthritis (JIA). In the box plots, the boxes represent the interquartile range, the thick horizontal line within the boxes represents the median, and the whiskers extend to the most extreme data points (range).

The median concentrations of synovial fluid HMGB1 in patients with oligoarticular and polyarticular JIA (43477,16 and 42630,17 pg/ml) were higher than in serum (3551,97 pg/ml). In addition, the synovial fluid level of sRAGE were lower in both oligoarticular JIA and polyarticular JIA groups (234,68 and 84,52 pg/ml, respectively) than in serum (2269,84 and 1757,76 pg/ml, respectively) (Figure 2).

### Relationship between serum HMGB1 and sRAGE levels and common inflammatory indicators of JIA and SLE

We were assessing the correlation of the HMGB1 and sRAGE with the laboratory tests commonly used to evaluate the disease activity of JIA and SLE.

**Figure 3:**
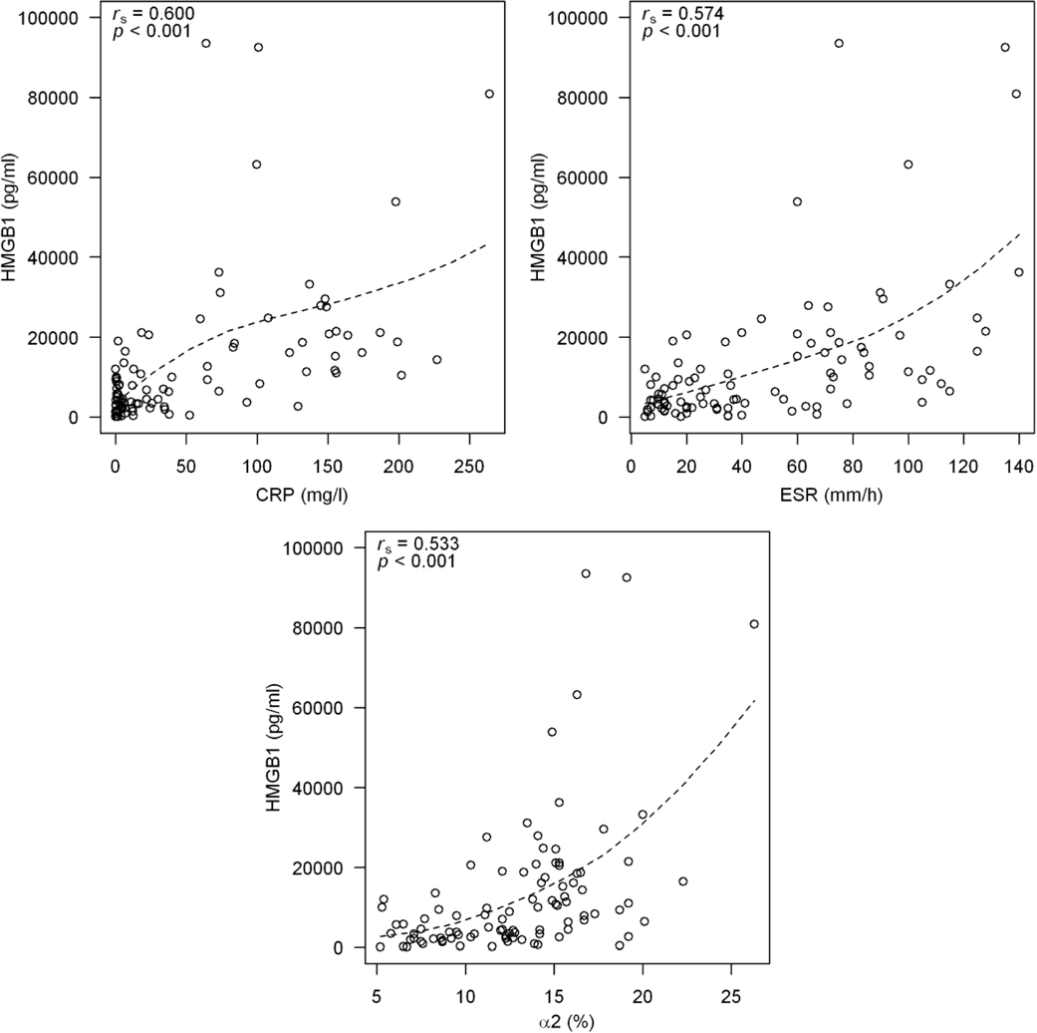
**Association of HMGB1 levels and ordinary acute-phase reactants in JIA children.** The positive correlations between serum high mobility group box-1 (HMGB1) levels and C-reactive protein (CRP), erythrocyte sedimentation rate (ESR), α2 globulins (α2) in 97 patients with juvenile idiopathic arthritis (JIA) were found. Spearman’s correlation coefficients (r) and p-values are given in each chart’s top left corner, a locally weighted polynomial regression line (loess) was super imposed on data points.

In patients with JIA, regardless of the disease type, there was statistically significant and positive correlation between serum levels of HMGB1 and ESR (r = 0,574, P < 0.001), CRP (r = 0,600; P < 0.001) and α2-globulin (r = 0,533; P < 0.001) (Figure 3) while weak statistically significant and negative correlation was found between HMGB1 and Hb (r = - 0.362; P < 0.002), Hct (r = - 0.322; P = 0.008) and RBC (r = - 0.285; P = 0.029).

In all patients with JIA, levels of sRAGE exhibit weak but statistically significant negative correlation with the levels of CRP (r = - 0,283; P = 0.010), and ESR (r = - 0,347; P = 0.001).

**Figure 4:**
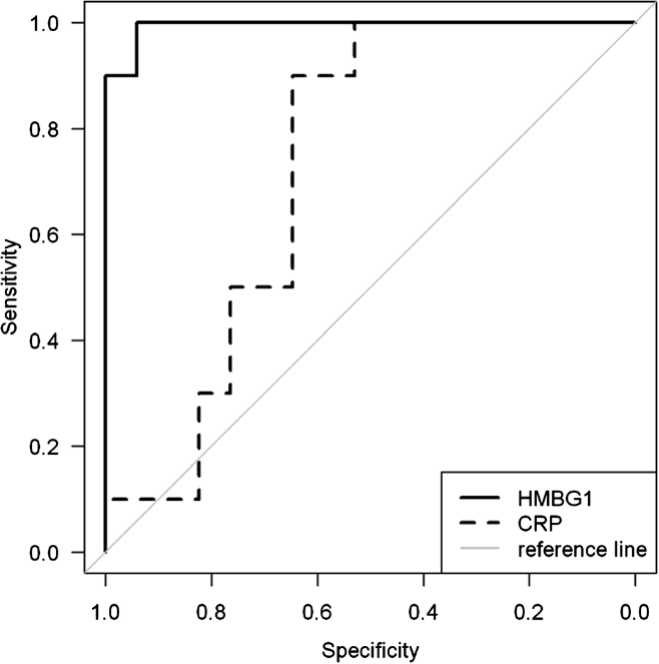
**Receiveroperatingcharacteristiccurveof HMGB1 and C-reactive protein (CRP) serum levels insystemiconset JIA.** Among the participants with systemic onset JIA, levels of HMGB1 and CRP were significantly higher in patients with hepatosplenomegaly and serositis compared to those without these clinical characteristics. HMGB1 was more sensitive than CRP in detecting hepatosplenomegaly and serositis in these patients, as shown by receiver operating characteristics (ROC) curve. The area under the curve for HMGB1 was 0.9412, the mean ± 95% confidence, using a cut off value of 19595 pg/ml for HMGB1 and136 mg/l for CRP.

Additionally, in children with SLE no association was detected between HMGB1 or sRAGE to the commonly used laboratory tests, but there was statistically significant and positive correlation between sRAGE levels and C4 complement component (r =0.47, P <0.045). Among the study participants with systemic onset JIA, levels of HMGB1 were significantly higher in patients with hepatosplenomegaly and serositis compared to those without these clinical characteristics (27695,03 and 12678,06 pg/ml; P < 0.001). The CRP levels of systemic JIA patients presenting with hepatosplenomegaly or serositis were also significantly higher but HMGB1 was more sensitive than CRP in detecting hepatosplenomegaly or serositis in these patients, as shown by receiver operating characteristics (ROC) curve assessment. The area under the curve (AUC) for HMGB1 was 0.9941 and for CRP was 0.7294 (mean with 95% confidence interval). At the optimal cut-off value of 19595 pg/ml for HMGB1, the diagnostic sensitivity was 94% and specificity was 100%. At a cut-off concentration of 136 mg/l CRP revealed a sensitivity of 64% and a specificity of 100% (Figure 4).

## Discussion

Our study points to the deregulation of the alarmin system in both JIA and juvenile SLE, demonstrated through a significant increase of the serum levels of HMGB1, coupled with a decrease of its decoy receptor, soluble RAGE. Although previous studies have implicated that HMGB1 and sRAGE are involved in and considered as biomarkers for a number of inflammatory diseases [14–16], reports on JIA and juvenile SLE are quite scarce [13, 26–29].

Antibodies to HMGB1 protein were detected in sera of children with JIA although the relevance of that finding still remains to be unveiled [26, 27]. Moreover, recent research has recorded high levels of HMGB1 in synovial fluid in children with JIA correlating with early onset of disease [28]. In addition, there is one in vivo study in cSLE patients that has shown a correlation of HMGB1 with the disease activity regardless of the presence of nephritis [29].

HMGB1, has also been evaluated as one of neutrophil proteins in cSLE. Upon stimulation with anti-RNP antibodies, mature SLE neutrophils while dying release NETs containing much of HMGB1 [13]. The previous observations implicated the role of HMGB1 on the JIA and cSLE pathogenesis.

In the present work, we found significantly higher HMGB1 levels in all subtypes of JIA patients versus healthy controls, with the levels of HMGB1 particularly elevated in children with sJIA. Furthermore, the levels of HMGB1 were the highest in the subgroup of sJIA patients with hepatosplenomegaly or serositis, which is in accordance with the known release of HMGB1 in acute systemic inflammatory conditions such as sepsis [36]. Our data point to systemic release of HMGB1 as a possible trigger for uncontrolled inflammatory activity in sJIA. The result of elevated serum HMGB1 in children with SLE mirrors the situation in SLE adults, where HMGB1 has been implicated in the pathogenesis and manifestations of the disease. For example, HMGB1 is often detectable in the skin lesions of patients with chronic cutaneous lupus and may also act as a proinflammatory mediator in antibody-induced kidney damage in SLE [37–39]. Since HMGB1 is an essential part of the immune complexes in lupus and since the immune complexes lead to immune activation via a pathway involving RAGE, the elevation of HMGB1 and the simultaneous decline of sRAGE could favour HMGB1-RAGE signalling as an important pathogenic mechanism.

Interestingly, our patients presenting with lupus nephritis were also characterized by high levels of HMGB1, with statistical significance. It could be, therefore, possible that HMGB1 may act as a proinflammatory mediator in kidney damage in SLE, as shown previously [40]. An inverse relationship between HMGB1 and its decoy receptor, sRAGE, in the serum of children with JIA and in SLE was not a surprise. Lower serum levels of sRAGE are in the line with a report on decreased levels of sRAGE in chronic inflammatory diseases [24, 25]. Although it is still not clear whether the high levels of HMGB1 cause diminishing level of sRAGE by the consumption of this soluble receptor or whether low levels of sRAGE strengthen proinflammatory effect of HMGB1, a study by Schaper et al. demonstrated that the increased serum levels of HMGB1 in patients with SLE lead to the consumption of sRAGE during the inflammatory process [41]. In contrast to these studies, there is one work which has shown higher level of sRAGE in active SLE [42]. The result disparity is possible due to a small number of subjects, only 10, and as pointed out by the authors of the aforementioned study, the cause may be in medication use as well.

Further, we aimed to investigate the potential role of HMGB1 as a marker of disease activity with JIA and SLE serving as a model of acute inflammation. It is established that ESR and CRP, classic markers of inflammation are raised in sJIA, but it is not specific for the detection of sJIA activity. In our paper a significant positive correlation was observed between HMGB1 and ESR, CRP and α2 globulin which suggests that the measurement of serum HMGB1 protein levels in serum may be a useful tool in the evaluation of sJIA patients, at least in acute inflammation. This is corroborated by the fact that although the CRP levels in systemic JIA patients presenting with hepatosplenomegaly or serositis were very high, HMGB1 was a more sensitive indicator (a diagnostic sensitivity for HMGB1 was 94% while for CRP was 64%). It is well known that HMGB1 is released at the site of joint inflammation and that the injection of HMGB1 into a normal joint causes development of arthritis [43–45]. In that context, our finding of increased amounts of HMGB1 in inflamed joints of children with oligoarticular and polyarticular JIA led us to the hypothesis that the another possible origin of HMGB1 in JIA could have been the inflamed joints and that serum HMGB1 was not synthesised only by the circulating leukocytes. However, a further research, aimed at elucidating the exact role of the alarmin system, is obviously called for. A possible shortcoming of our study was the small number of SLE patients. However we would like to point out that we were dealing with the disease which in not considered common among pediatric patients. For instance, the overall annual incidence rate of Croatian children with SLE varied from 1 to 15 per million children in the last 20 years [46].

## Conclusion

We believe that our description of an inverse relationship of increased serum levels of HMGB1 and decreased serum levels of sRAGE in blood samples taken at diagnosis indicates that inflammation triggered by alarmins plays a role in pathogenesis of JIA and juvenile SLE. Furthermore, the results presented here suggest that alarmin may be a potential therapeutic target for immunotherapy of JIA and SLE, but also suggest that HMGB1 may be a valuable laboratory biomarker. Further studies in longitudinally collected blood samples over time from patients may reveal whether HMGB1 could be a valuable biomarker even more sensitive and specific than other available indicators of inflammation.

## Abbreviations

JIA: Juvenile idiopathic arthritis
RA: Rheumatoid arthritis
SLE: Systemic lupus erythematosus
HMGB1: High mobility group box-1
NET: Neutrophil extracellular traps
sRAGE: Soluble receptor for advanced glycation end-products
TLRs: Toll-like receptors
CXCR4: Chemokine receptor C-X-C motif receptor 4
sRAGE: Soluble receptor for advanced glycation end-products
ELISA: Enzyme-linked immunosorbent assay.
